# K_2*x*_Sn_4–*x*_S_8–*x*_ (*x* = 0.65–1): a new metal sulfide for rapid and selective removal of Cs^+^, Sr^2+^ and UO_2_^2+^ ions†
†Electronic supplementary information (ESI) available: Raman spectra, thermogravimetric analysis, scanning electron microgram, X-ray crystallographic file (CIF) containing crystallographic refinement details, atomic coordinates with equivalent isotropic displacement parameters, anisotropic displacement parameters, and selected bond distances for KTS-3. See DOI: 10.1039/c5sc03040d


**DOI:** 10.1039/c5sc03040d

**Published:** 2015-10-27

**Authors:** Debajit Sarma, Christos D. Malliakas, K. S. Subrahmanyam, Saiful M. Islam, Mercouri G. Kanatzidis

**Affiliations:** a Department of Chemistry , Northwestern University , 2145 Sheridan Road , Evanston , IL 60208 , USA . Email: m-kanatzidis@northwestern.edu; b Materials Science Division , Argonne National Laboratory , Argonne , IL 60439 , USA

## Abstract

The fission of uranium produces radionuclides, ^137^Cs and ^90^Sr, which are major constituents of spent nuclear fuel. The half-life of ^137^Cs and ^90^Sr is nearly 30 years and thus that makes them harmful to human life and the environment. The selective removal of these radionuclides in the presence of high salt concentrations from industrial nuclear waste is necessary for safe storage. Here we report the synthesis and crystal structure of K_2*x*_Sn_4–*x*_S_8–*x*_ (*x* = 0.65–1, KTS-3) a material which exhibits excellent Cs^+^, Sr^2+^ and UO_2_^2+^ ion exchange properties in varying conditions. The compound adopts a layered structure which consists of exchangeable potassium ions sandwiched between infinite layers of octahedral and tetrahedral tin centers. K_2*x*_Sn_4–*x*_S_8–*x*_ (*x* = 0.65–1, KTS-3) crystallizes in the monoclinic space group *P*2_1_/*c* with cell parameters *a* = 13.092(3) Å, *b* = 16.882(2) Å, *c* = 7.375(1) Å and *β* = 98.10(1)°. Refinement of the single crystal diffraction data revealed the presence of Sn vacancies in the tetrahedra that are long range ordered. The interlayer potassium ions of KTS-3 can be exchanged for Cs^+^, Sr^2+^ and UO_2_^2+^. KTS-3 exhibits rapid and efficient ion exchange behavior in a broad pH range. The distribution coefficients (*K*_d_) for KTS-3 are high for Cs^+^ (5.5 × 10^4^), Sr^2+^ (3.9 × 10^5^) and UO_2_^2+^ (2.7 × 10^4^) at neutral pH (7.4, 6.9, 5.7 ppm Cs^+^, Sr^2+^ and UO_2_^2+^, respectively; *V*/*m* ∼ 1000 mL g^–1^). KTS-3 exhibits impressive Cs^+^, Sr^2+^ and UO_2_^2+^ ion exchange properties in high salt concentration and over a broad pH range, which coupled with the low cost, environmentally friendly nature and facile synthesis underscores its potential in treating nuclear waste.

## Abstract 2

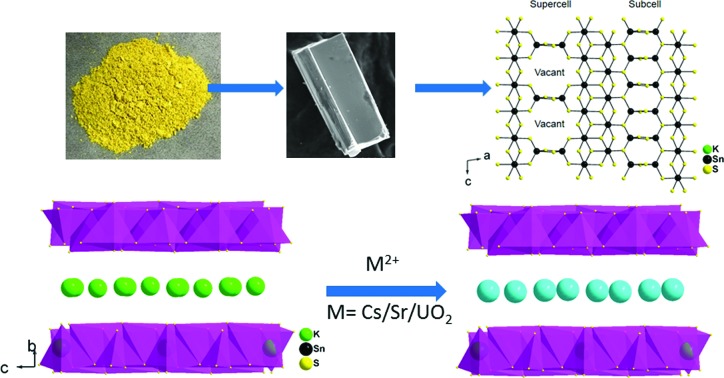
The synthesis and crystal structure of K_2*x*_Sn_4–*x*_S_8–*x*_ (*x* = 0.65–1, KTS-3) a material which exhibits excellent Cs^+^, Sr^2+^ and UO_2_^2+^ ion exchange properties in varying conditions are reported.

## Introduction

The pursuit of efficient, cheap, sustainable and growing sources of energy, involves nuclear energy which has emerged as one of the prominent alternatives in many countries and accounted for 12.3% of the world's electricity production in 2012.^1^ Over the last four decades the accumulation of radioactive spent nuclear fuel (nuclear waste) has reached a staggering volume of 71 780 metric tons and it is increasing by 2300 metric tons every year.^2^ The rapidly increasing number of nuclear power plants will generate even larger amounts of nuclear waste. The prime source of nuclear fuel is various uranium salts, which are being used in different stages from mining, nuclear fabrication and processing. The uranium salts form a major component of the nuclear waste along with the fission generated non-actinide isotopes. The estimated amount of uranium present in seawater is 4 × 10^12^ kg (at ∼3 ppb), so potentially it could supply nuclear fuel for thousands of years.^3^ The primary issue with isolation of uranium from seawater in a cost effective manner is the presence of other ions (Na^+^, Cl^–^, Mg^2+^, SO_4_^2–^, Ca^2+^, and CO_3_^2–^) in predominant amounts. ^90^Sr and ^137^Cs are the main hazardous fission generated non-actinide isotopes present in nuclear waste, as they produce gamma and high energy beta particles.^4^^90^Sr (with a half-time of *t*_1/2_ ∼ 29 years) and ^137^Cs (*t*_1/2_ ∼ 30 years) pose a major long-term risk due to their long half-life. Recently, the tsunami-induced disaster at the Fukushima nuclear power plant in 2011 resulted in contamination of a wide region of the northern Kanto and Tohoku areas in Japan with radionuclides, ^131^I, ^134^Cs, ^137^Cs, and ^90^Sr.^5^–^7^ Therefore, nuclear waste needs to be dealt with effectively, for safe storage and disposal due to its adverse health effects in humans and the environment.

The most commonly used technique for the separation of radioactive elements from industrially produced nuclear waste is solvent extraction using liquid phase organic compounds.^8^–^10^ The use of ion exchange media is another alternative for the removal of radionuclides from the nuclear waste,^11^–^22^ however, they are relatively less explored due to certain drawbacks: the organic ion exchange materials are efficient but costly, whereas the inorganic ion exchange materials are cheaper but they are less efficient because of low selectivity for the ions of interest. So, there is a growing need to develop efficient inorganic ion exchange materials for radioactive species.

Over the past decade or so metal sulfides have emerged as a selective class of ion exchangers for capturing soft metal ions such as Hg, Cd, Ag *etc.*^20^,^23^,^24^ Chalcogenide open-framework compounds, such as K_6_Sn[Zn_4_Sn_4_S_17_]^17^ and (NH_4_)_4_In_12_Se_20_ (ref. 19) present unique advantages over their oxide analogues. The layered thiostannates are particularly interesting because they exhibit open accessible structures where ion-exchange chemistry can occur readily.^25^–^30^ In previous work, we proposed that layered metal sulfides K_2*x*_M_*x*_Sn_3–*x*_S_6_ (M = Mn, KMS-1; M = Mg, KMS-2) can be used for facile ion exchange of Sr^2+^, Cs^+^ and UO_2_^2+^.^18^,^19^,^31^,^32^ A variety of synthetic parameters were explored in the search for new compounds based on a tin sulfide layer structure to modulate the ion exchange properties. The advantage of the chalcogenide materials stems from the fact that they are based on softer chalcogen ligands (in the Lewis base sense) which can induce high selectivity for heavy metal ions, Cs^+^, Sr^2+^, UO_2_^2+^ against co-present hard ions such as Na^+^, Al^3+^ and Ca^2+^.^17^,^19^,^31^–^33^

Herein, we report a new ternary layered compound, K_2*x*_Sn_4–*x*_S_8–*x*_ (*x* = 0.65–1, KTS-3) and its promising selectivity for removing Cs^+^, Sr^2+^ and UO_2_^2+^ species *via* ion exchange processes. Specifically, we find that KTS-3 exhibits high distribution coefficients (*K*_d_) for the capture of Cs^+^ (5.5 × 10^4^), Sr^2+^ (3.9 × 10^5^) and UO_2_^2+^ (2.7 × 10^4^) over a broad pH range (*V*/*m* ∼ 1000 mL g^–1^). We find that KTS-3 remains highly effective for these ions even in presence of a large amount of Na^+^ ions.

## Experimental section

### Starting materials

KTS-3 was synthesized using high purity K_2_CO_3_ (99%, Sigma-Aldrich), tin powder (<150 μm, 99.5%, Sigma-Aldrich) and elemental sulfur (5N Plus Inc.).

### Hydrothermal synthesis of K_2*x*_Sn_4–*x*_S_8–*x*_ (*x* = 0.65–1, KTS-3)

**Fig. 1:**
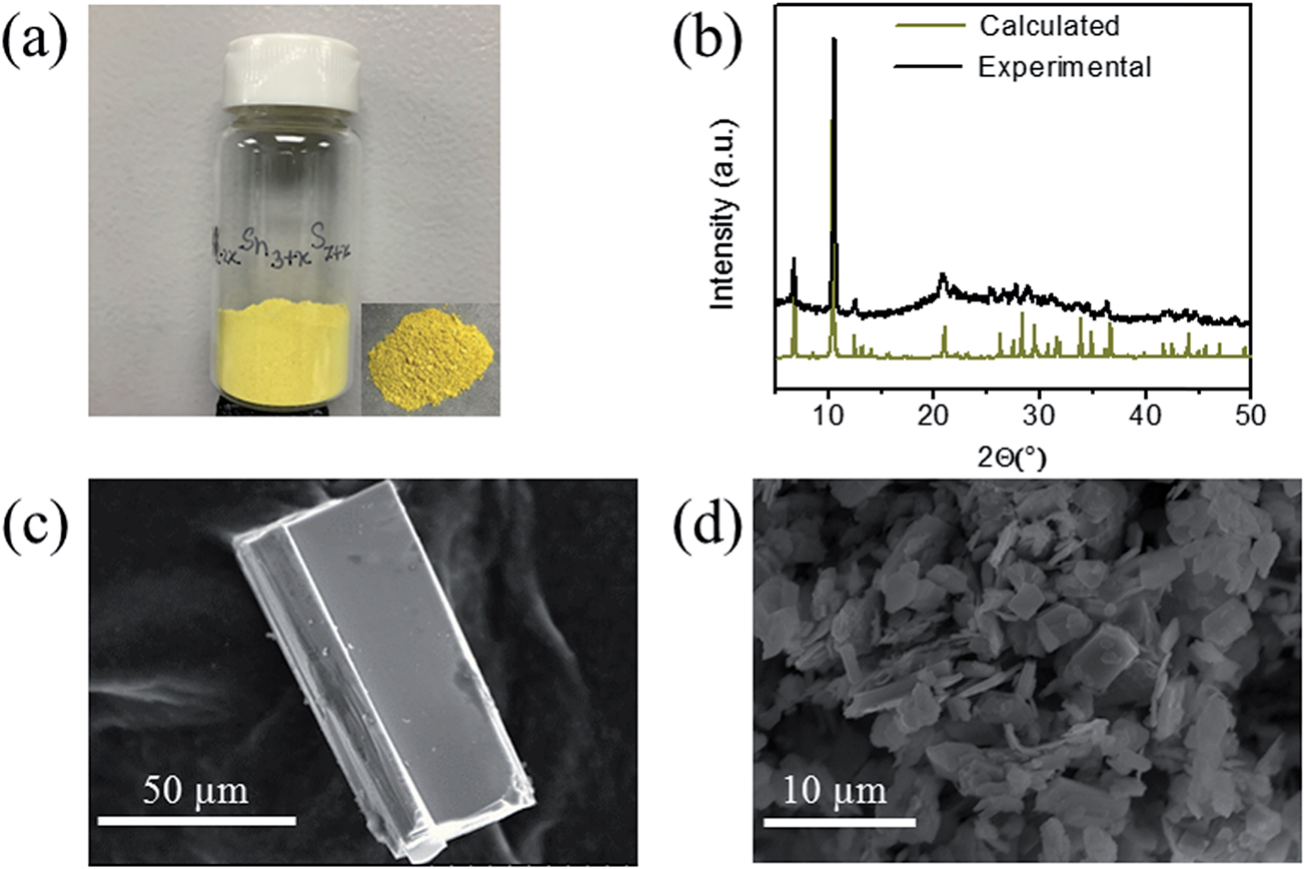
(a) Typical appearance of the KTS-3 sample, (b) the calculated and the experimental PXRD pattern, (c) SEM image of a KTS-3 crystal and (d) SEM image of the polycrystalline powder of K_2*x*_Sn_4–*x*_S_8–*x*_ (*x* = 0.65–1, KTS-3).

K_2_CO_3_ (6 mmol, 0.830 g), elemental Sn (9 mmol, 1.068 g), S (30 mmol, 0.962 g) were taken in a 23 mL polytetrafluoroethylene (PTFE) lined stainless steel autoclave and deionized water (0.5 mL) was added drop wise until the mixture acquired dough-like consistency. The autoclave was sealed properly and maintained in a preheated oven at 220 °C for 15 h under autogenous pressure. Then, the autoclave was allowed to cool to room temperature. The product was found to contain yellow rod shaped crystals along with yellow polycrystalline powder (Fig. 1a). The product was isolated by filtration, washed several times with water, acetone and dried under vacuum. The yield was ∼2.0 g (∼85%, based on Sn) and the product was air and moisture stable. Electron Dispersive Spectroscopy (EDS) analysis shows the presence of K, Sn and S and gave an average formula “K_1.34_Sn_3.26_S_7.32_”.

### Ion-exchange experiments

A typical ion exchange experiment of KTS-3 with Cs^+^, Sr^2+^, or UO_2_^2+^ was carried out in a 20 mL scintillation vial, where measured amounts of CsCl, SrCl_2_·6H_2_O or UO_2_(NO_3_)·6H_2_O (0.1 mmol of A^*n*+^) were dissolved in deionized water (10 mL) and KTS-3 (40 mg) was added. Then the mixture was kept under magnetic stirring for anywhere from 5 min to 15 h at room temperature. The ion exchanged material was centrifuged and isolated by filtration (through filter paper, Whatman no. 1), washed several times with water and acetone, and dried under vacuum. In all cases, the ion exchange reaction was completed after only one cycle (EDS showed all potassium ions were exchanged).

The distribution coefficient *K*_d_, used for the determination of the affinity and selectivity of KTS-3 for Cs^+^, Sr^2+^, or UO_2_^2+^ is given by the equation: *K*_d_ = (*V*/*m*)[(*C*_0_ – *C*_f_)/*C*_f_] where, *V* is the volume (mL) of the testing solution, *m* is the amount of the ion exchanger (g), *C*_0_ and *C*_f_ are the initial and equilibrium concentration of a given ion A^*n*+^ (ppm).

The individual Cs^+^, Sr^2+^, or UO_2_^2+^ uptake from solutions of various concentrations were studied with *V*/*m* ≈ 1000 mL g^–1^, at room temperature and 15 h contact time. The data obtained were used for the determination of the sorption isotherms. All ion exchange experiments reported in this work were performed by the batch method in 20 mL scintillation vials.

The individual ion exchange experiments for the Cs^+^, Sr^2+^, or UO_2_^2+^ ions at different pH and salt concentration were also carried out. The required pH values (2, 4, 6, 8, 10 and 12) were achieved by diluting the commercial standards (1000 ppm) with HCl or NaOH solution to ∼6 ppm. The ion exchange experiment at different Na^+^ concentration was done by dissolving the required amount of NaCl in 10 mL solution of A^*n*+^ ion (∼6 ppm). The exchange experiments were performed with *V*/*m* ≈ 1000 mL g^–1^, at room temperature and 15 h contact.

Competitive ion exchange (Cs^+^ and Sr^2+^) experiments of KTS-3 were also carried out with a *V*/*m* ratio of 1000 mL g^–1^, at room temperature with 15 h of contact time. The initial concentration was approximately ∼6 ppm for both the ions. The competitive ion exchange experiments were similar to those of the individual ion exchange experiments except they contained both Cs^+^ and Sr^2+^ ions in solution.

The kinetic studies of the adsorption of ions by KTS-3 were carried out as follows: ion-exchange experiments of various reaction times (5, 15, 30, 60, 120, 300 and 1200 min) were performed. For each experiment, 10 mg of KTS-3 was weighed into a 20 mL vial. A 10 mL sample of water solution containing ∼1 ppm of Cs^+^/Sr^2+^/UO_2_^2+^ was added to each vial, and the mixtures were kept under magnetic stirring (pH ∼ 7). The suspensions were filtered after the designated reaction time and the filtrates were analyzed by inductively coupled plasma-mass spectroscopy (ICP-MS).

### Powder X-ray diffraction

The powder X-ray diffraction (PXRD) patterns were collected at room temperature with a CPS 120 INEL X-ray powder diffractometer with graphite monochromated Cu Kα radiation operating at 40 kV and 20 mA. The samples were prepared by grinding and spreading over a glass slide.

### Single-crystal X-ray crystallography

A suitable single crystal was carefully selected under a polarizing microscope and glued to a thin glass fiber. Single crystal data were collected on a STOE IPDS II diffractometer using Mo Kα radiation (*λ* = 0.71073 Å) at room temperature. The generator was operated at 50 kV and 40 mA. The data were collected with a ω scan width of 1° keeping the crystal to detector distance fixed at 8.0 cm. Integration and numerical absorption corrections were performed using X-AREA, X-RED, and X-SHAPE.^34^ The structure was solved using direct methods and refined by the SHELXTL program package^35^ using a full-matrix least squares refinement against the square of structure factors. Final structure refinement included atomic positions and anisotropic thermal parameters for all Sn and S atoms. The thermal displacement parameters of the disordered K atoms was refined isotropically. Details of the structure solution and final refinements for the compound are given in Table 1.

**Table 1:** Crystal data and structure refinement at room temperature for the subcell and supercell of K_1.92_Sn_3.04_S_7.04_^*a*^

K_2_Sn_3_S_7_	Subcell	Supercell
Formula weight	658.69	661.31
Wavelength	0.71073 Å	
Crystal system	Orthorhombic	Monoclinic
Space group	*Cmcm*	*P*2_1_/*c*
Unit cell dimensions	*a* = 3.6831(2) Å, *α* = 90°	*a* = 13.092(3) Å, *α* = 90°
	*b* = 25.8877(19) Å, *β* = 90°	*b* = 16.882(2) Å, *β* = 98.100(15)°
	*c* = 16.8155(11) Å, *γ* = 90°	*c* = 7.3748(13) Å, *γ* = 90°
Volume	1603.31(18) Å^3^	1613.7(5) Å^3^
*Z*	4	
Density (calculated)	2.729 g cm^–3^	2.722 g cm^–3^
Absorption coefficient	6.026 mm^–1^	6.029 mm^–1^
*F*(000)	1200	1204
Color	Yellow	
Crystal size	0.320 × 0.160 × 0.050 mm^3^	
Index ranges	–4 ≤ *h* ≤ 4, –34 ≤ *k* ≤ 34, –22 ≤ *l* ≤ 22	–19 ≤ *h* ≤ 19, –25 ≤ *k* ≤ 25, –10 ≤ *l* ≤ 11
Reflections collected	13 142	37 136
Independent reflections	1145 [*R*_int_ = 0.0590]	5540 [*R*_int_ = 0.094]
Completeness to *θ* = 25.242°	99.7%	100.0%
Refinement method	Full-matrix least-squares on *F*^2^	
Data/restraints/parameters	1145/0/45	5540/1/121
Goodness-of-fit	1.140	1.135
Final *R* indices [*I* > 2*σ*(*I*)]	*R*_obs_ = 0.0749, *wR*_obs_ = 0.2183	*R*_obs_ = 0.1063, *wR*_obs_ = 0.2807
*R* indices [all data]	*R*_all_ = 0.0796, *wR*_all_ = 0.2237	*R*_all_ = 0.1410, *wR*_all_ = 0.3100
Extinction coefficient		0.0019(5)
Largest diff. peak and hole	3.868 and –1.905 e Å^–3^	4.342 and –2.783 e Å^–3^
Weighting scheme	*a* = 0.1110, *b* = 74.4974	*a* = 0.141, *b* = 21.5465

^*a*^ *R* = ∑‖*F*_o_| – |*F*_c_‖/∑|*F*_o_|, *wR* = {∑[*w*(|*F*_o_|^2^ – |*F*_c_|^2^)^2^]/∑[*w*(|*F*_o_|^4^)]}^1/2^ and *w* = 1/[*σ*^2^(*F*_o_^2^) + (*aP*)^2^ + *bP*] where *P* = (*F*_o_^2^ + 2*F*_c_^2^)/3. The twin law [1 0 0.5 0 –1 0 0 0 –1] was used with a refined fraction of 45.0(3)%.

### Scanning electron microscopy and energy dispersive spectroscopy

The energy dispersive spectroscopy (EDS) was performed with a Hitachi S-3400N-II scanning electron microscope (SEM) equipped with an ESED II detector. An accelerating voltage of 20 kV and 60 seconds acquisition time were used for elemental analysis.

### Thermogravimetric analysis

The thermogravimetric analysis (TG) was performed with a Shimadzu TGA-50 system under nitrogen atmosphere in an aluminum crucible. The analysis was performed with a heating rate of 10 °C min^–1^ and a nitrogen flow rate of 40 mL min^–1^ from room temperature to 600 °C.

### Differential thermal analysis

The differential thermal analyses (DTA) were performed on a Shimadzu DTA-50 thermal analyzer. For a typical analysis, around 30 mg of sample was sealed in a quartz ampoule and sealed under vacuum, another sealed quartz ampoule with Al_2_O_3_ was used as reference material. The analysis was performed with a heating rate of 2 °C min^–1^ and a nitrogen flow rate of 30 mL min^–1^ from room temperature to 600 °C.

### Infrared (IR) and Raman spectroscopy

Infrared spectra of compounds were collected on a Bruker Tensor 37 FTIR (MID IR/ATR) using an attenuated total reflectance attachment in the range 4000–600 cm^–1^. The Raman spectra of the ground samples were collected on a DeltaNu Raman system that uses a 785 nm constant wavelength laser. The spectra were collected in the range of 100–2000 cm^–1^ with the sample inside a 0.5 mm capillary tube.

### Band gap measurements

The UV-vis/near-IR diffuse reflectance spectra of the ground samples were collected using a Shimadzu UV03010 PC double beam, double monochromator spectrophotometer in the wavelength range of 200–2500 nm. BaSO_4_ powder was used as a reference and base material on which the powder sample was coated. Using the Kubelka–Munk^36^ equation the reflectance data were converted to absorption data and the band edge of the sample was calculated from the intercept of the line extrapolated from the high energy end to the baseline.

### X-ray photoelectron spectroscopy (XPS) analysis

XPS of the KTS-3 and exchanged materials were performed on ground powders using a Thermo Scientific ESCALAB 250 Xi spectrometer equipped with a monochromatic Al Kα X-ray source (1486.6 eV) operating at 300 W. Samples were analyzed under vacuum (*P* < 10^–8^ mbar) with a pass energy of 150 eV (survey scans) and 25 eV (high-resolution scans). A low-energy electron flood gun was employed for charge neutralization. Ion beam etching was performed to clean off some of the surface contamination. Prior to the XPS measurements, the crystalline powders were pressed on copper foil and mounted on stubs and successively put into the entry-load chamber to pump. All peaks were referenced to the signature C1s peak binding energy at 284.6 eV for adventitious carbon. Avantage software was used to fit the experimental peaks.

### Inductively coupled plasma-mass spectroscopy

The Cs^+^, Sr^2+^, and UO_2_^2+^ ion exchange samples and the competitive ion exchange samples (Cs^2+^ and Sr^2+^) were analyzed with Inductively Coupled Plasma-Mass Spectroscopy (ICP-MS) using a computer-controlled ThermoFisher X Series II Inductively Coupled Plasma Mass Spectrometer with a quadruple setup equipped with Collision Cell Technology. Eleven standards were prepared in the range of 0.78–800 ppb by diluting commercial solutions (Sigma-Aldrich). The ion exchange samples were diluted to lower the concentrations below 800 ppb. All the samples and standards were prepared in a 5% (nitric acid + hydrochloric acid) solution with 1 ppb (Bi, Ho, In, Li, Tb, Y) internal standard in order to correct the instrumental drift and matrix effects during analysis.

## Results and discussion

### Synthesis and characterization

The synthesis of K_2*x*_Sn_4–*x*_S_8–*x*_ (*x* = 0.65–1, KTS-3) was accomplished by a hydrothermal method at 220 °C. The product was found to contain a large amount of yellow powder along with few rod shaped yellow crystals. The powder X-ray diffraction of the samples of KTS-3 showed that the yellow powder and the crystals are the same material and confirmed the phase purity (Fig. 1b) when compared against the calculated pattern obtained by the single crystal model. The product was also analyzed with semi-quantitative SEM-EDS (Fig. 1c and d) which showed the presence of K, Sn, S and revealed an average composition of K_1.34_Sn_3.26_S_7.32_. The value of *x* = 0.65–1 was determined by analyzing different sets of samples with SEM-EDS and ICP-MS. The single crystal data of the rod shaped crystals revealed a layer structure of composition K_1.92_Sn_3.04_S_7.04_. The Raman spectra of the KTS-3 sample shows three sharp bands at 321, 355 and 382 cm^–1^ and a small band at 247 cm^–1^. The bands at 321, 355 and 382 cm^–1^ are consistent with octahedral and tetrahedral Sn–S bond vibrations (Fig. S1a†).^37^,^38^ The 247 cm^–1^ band may arise from collective lattice modes or from the vibrations associated with the potassium ions.

Thermogravimetric (TG) analysis of the KTS-3 compound was carried out in flowing nitrogen gas (flow rate = 20 mL min^–1^) in the temperature range 20–600 °C (heating rate of 10 °C min^–1^). The TG studies indicate that KTS-3 exhibits a single-step weight loss of ∼10% up to 235 °C which corresponds to the loss of adsorbed water molecules. The compound remains stable up to 525 °C, after which it starts to decompose (Fig. S1b†) into K_2_Sn_2_S_5_ and SnS_2_ as determined by powder XRD. Differential thermal analysis (DTA) of the samples shows no sign of melting up to 600 °C (Fig. S2†).

**Fig. 2:**
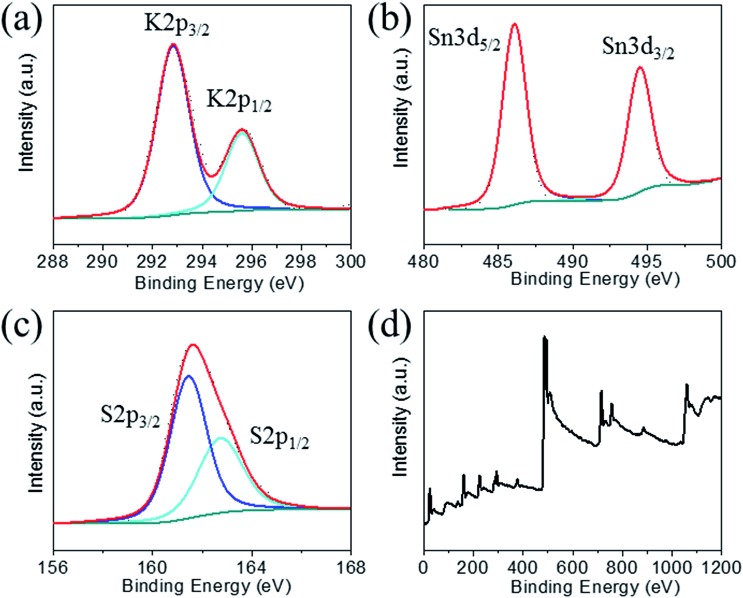
X-ray photoelectron spectra of (a) potassium, (b) tin, (c) sulfur, and (d) survey spectrum for KTS-3. Dotted and solid lines represent experimental and deconvoluted spectra, respectively.

X-ray photoelectron spectroscopy performed on KTS-3 (Fig. 2) shows peaks at 292.5 and 295.6 eV which are characteristic for 2p_3/2_ and 2p_1/2_ of K^+^ cations.^39^ The peaks at 486.0 and 494.5 eV are consistent with the 3d_5/2_ and 3d_3/2_ levels observed for Sn^4+^ cations.^39^ The sulfur 2p orbital excitations appear as a broad peak in the range 158–165 eV. The deconvolution of the broad band gives two bands centered at 161.5 and 162.7 eV which are characteristic of 2p_3/2_ and 2p_1/2_ sulfide anions, respectively.^39^,^40^

### Crystal structure

**Fig. 3:**
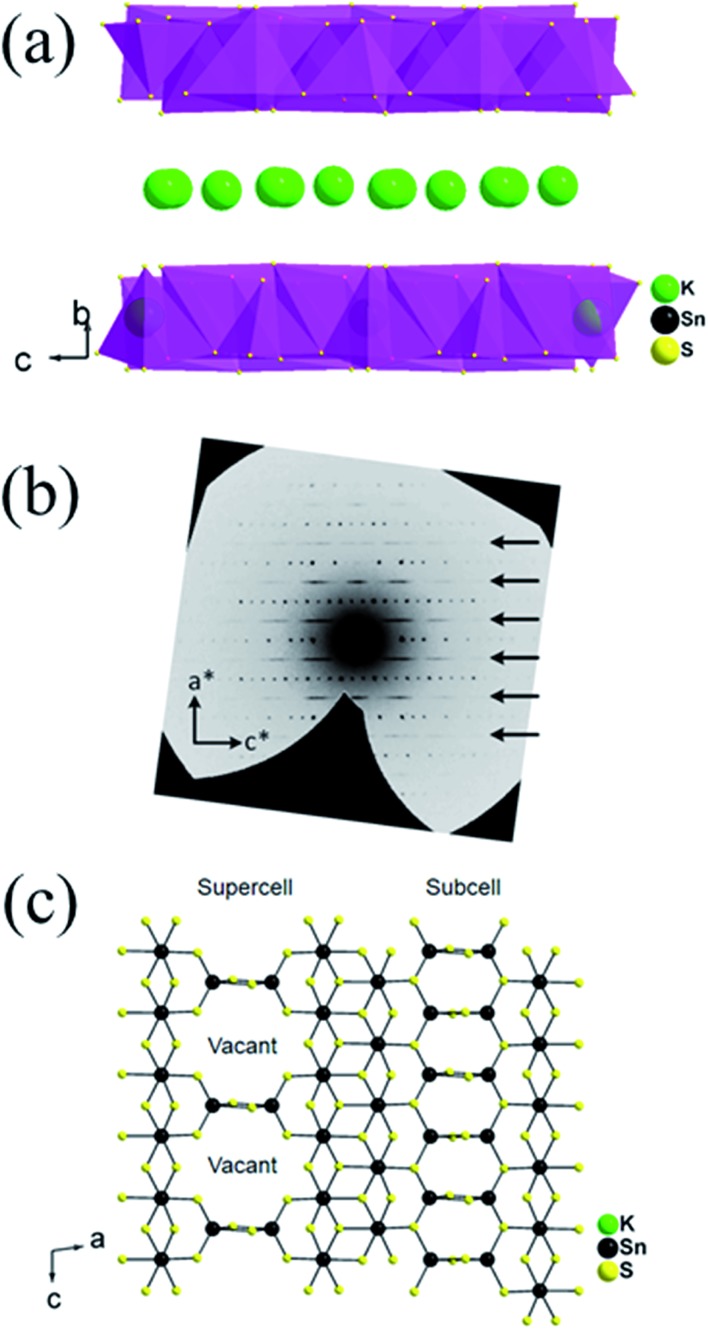
(a) The layer structure of K_1.92_Sn_3.04_S_7.04_, (b) synthetic precession image of the (*h*5*l*) layer with the diffuse super cell reflections shown with arrows, and (c) [Sn_3_S_7_]^2–^ layer with ideally ordered Sn and vacancy sites. Refinement suggests the presence of some Sn atoms in the vacant sites with fractional occupancy of 31.1(5)% due to the diffuse character of the supercell reflections.

The structure of K_1.92_Sn_3.04_S_7.04_ is composed of infinite layers of [Sn_3_S_7_] stacked along the *b*-axis with K ions residing between the layers, Fig. 3a. The [Sn_3_S_7_] layer consists of [SnS_6_] octahedra and [SnS_4_] tetrahedra. Edge-shared [SnS_6_] octahedral units form ribbons that run infinitely along the *c*-axis and have a width of two octahedral units. The [SnS_6_] ribbons are interconnected by edge-shared [SnS_4_] tetrahedra in the form of [Sn_2_S_6_] bridges. Potassium atoms are disordered and sandwiched between the [Sn_3_S_7_] layers. An apparent C-centered orthorhombic cell with *a* = 3.6831(2) Å, *b* = 25.8877(19) Å, and *c* = 16.8155(11) Å can index most of the reflections but after careful examination of the reciprocal lattice (Fig. 3b) we found the presence of additional broad and diffuse reflections that could be indexed by doubling of the short *a*-axis with a transformed primitive monoclinic unit cell of *a* = 13.092(3) Å, *b* = 16.882(2) Å, *c* = 7.375(1) Å and *β* = 98.10(1)°. The origin of the supercell is due to partial long range ordering of vacancies in the [SnS_4_] slabs where every other [Sn_2_S_6_] unit is missing along the *c*-axis, Fig. 3c.

The orthorhombic cell can be refined in *Cmcm* with a stoichiometry of ‘K_2_Sn_4_S_8_’ but this is problematic because this composition does not charge balance assuming K^+^, Sn^4+^, and S^2–^ ions. Furthermore, the agreement factor for the ‘K_2_Sn_4_S_8_’ refinement was very high at ∼14.5% with large negative residual electron density around the Sn(2) and S(4) sites. Upon refinement of the occupancy of Sn(2) and S(4) (50% disorderly occupied) but omitting the supercell reflections, the agreement factor improved significantly (7.5%, see Table 1) and the refined composition becomes K_1.92_Sn_3.04_S_7.04_ which is charge balanced. By subsequently introducing the intensity of the supercell reflections into the refinement, an additional long range ordering of the vacancies in the Sn(2) and S(4) sites was found. The supercell of KTS-3 was solved using the monoclinic spacegroup *P*2_1_/*c* and twining was required for a successful refinement. A refined twin fraction of 45.0(3)% was determined using a twin law of 180 degrees rotation along the *c*-axis, Table 1. The final agreement factor is satisfactory given the very broad and diffuse nature of the supercell reflections, Fig. 3b.

The asymmetric unit of the KTS-3 supercell has 15 atoms. Four crystallographically independent Sn^4+^ atoms (two sites are partially occupied), eight sulfide atoms (two sites are partially occupied) and three K^+^ ions (two sites are partially disordered). The Sn(1) and Sn(2) ions are octahedrally coordinated by six sulfur atoms, and Sn(3) and Sn(4) atoms are tetrahedrally coordinated by four sulfur atoms. The [SnS_6_] and [SnS_4_] units are shared through S(5)/S(6) edges, the [SnS_6_] units are edge-shared through S(1)–S(2), and the [SnS_4_] units are edge-shared through S(7)/S(7) and S(8)/S(8) edges. The Sn(1), Sn(2) distorted octahedra have Sn–S distances in the range of 2.504(2)–2.621(2) Å and the Sn(3) distorted tetrahedral have Sn–S distances in the range of 2.288(2)–2.486(2) Å. Because of the partial ordering of vacancies, Sn(3) and Sn(4) are disordered with a refined fractional occupancy of 73.0(4) and 31.1(5)%, respectively. The same occupancy values were used for the S atoms that edge-share the [SnS_4_] tetrahedra, *i.e.*, the occupancy factor of S(7) and S(8) was constrained at 73.0(4) and 31.1(5)%, respectively. All K atoms have relatively large thermal factors which is characteristic for loosely bound intercalated atoms found in ion-exchanged materials.^17^,^18^,^32^ K(1A) and K(1B) are delocalized with an average disordered distance of 2.32(1) Å and fractional occupancy of 60.7(5) and 39.3(5)%, respectively where K(3) fully occupies its own site.

The basic difference between the structure of KTS-3 and that of so-called KMS structures which are also layered (K_2*x*_M_*x*_Sn_3–*x*_S_6_; M = Mn, KMS-1; M = Mg, KMS-2)^18^,^32^ is in the structure of the layers themselves. The layers of KMS-1 and KMS-2 are essentially derived from the SnS_2_ structure by replacing randomly some of the octahedral Sn^4+^ ions by either Mn^2+^ (KMS-1) or Mg^2+^ (KMS-2) ions, where all the Sn/M (M = Mn or Mg) ions occupy octahedral sites and the sulfur ions are three coordinated.^18^,^32^ However, in case of KTS-3 there are both octahedral and tetrahedral centers that are connected by three and two coordinated sulfur atoms to form the layer structure. Disordered potassium ions are located between the SnS_2_ or Sn_3_S_7_ layers, Fig. 3c.

### Ion-exchange of KTS-3 with Cs^+^, Sr^2+^ and UO_2_^2+^ ions

**Fig. 4:**
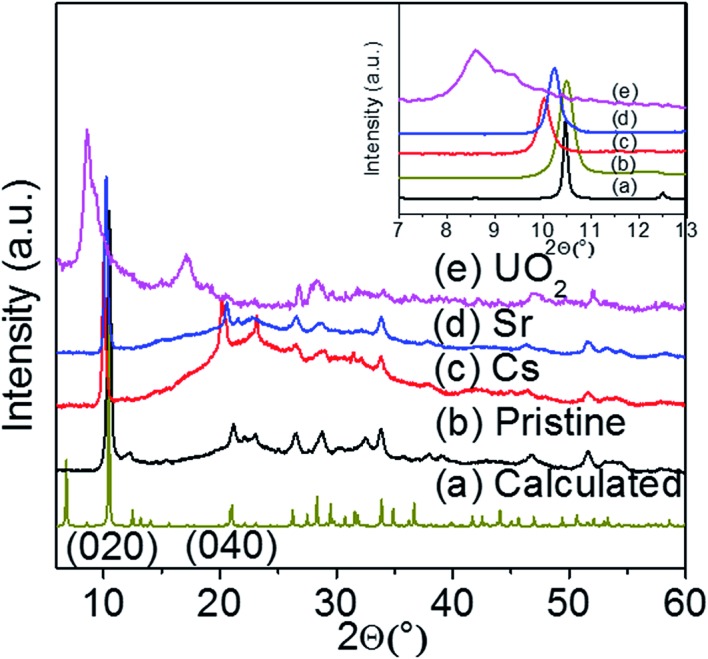
Powder X-ray diffraction patterns of pristine KTS-3 and the exchanged materials. The (020) and (040) reflection peaks for the exchanged materials shift towards lower 2*θ* (higher *d* spacing).

The interlayer potassium ions in the KTS-3 structure are disordered and move rapidly in an ion-exchange process. To check the feasibility of ion exchange of K^+^ in KTS-3 we immersed it in a solution of A^*n*+^ (A^*n*+^ = Cs^+^, Sr^2+^ and UO_2_^2+^) ions for 15 h. These ion exchange processes are in fact very rapid and almost all the ions were exchanged within 5 min, but to ensure a complete ion exchange we used 15 h. The EDS analysis of the materials after ion exchange showed the complete removal of the K^+^ ions. The EDS of the exchanged materials showed a ratio of 1.5 : 3 for Cs : Sn, 0.7 : 3 for Sr : Sn and 0.51 : 3 U : Sn, which are comparable with the expected Cs to Sn ratio (1.3–2.0) and Sr, UO_2_ to Sn ratio (0.65–1) (Fig. S3†). The PXRD of the exchanged materials showed isotactic ion exchange with retention of the parent structure (Fig. 4). The ion exchange processes can be described by the following equations:

1 K_1.92_Sn_3.04_S_7.04_ + 1.92CsCl → Cs_1.92_Sn_3.04_S_7.04_ + 1.92KCl

2 K_1.92_Sn_3.04_S_7.04_ + 0.96SrCl_2_·6H_2_O → [Sr(H_2_O)_*y*_]_0.96_Sn_3.04_S_7.04_ + 1.92KCl

3 K_1.92_Sn_3.04_S_7.04_ + 0.96UO_2_(NO_3_)_2_·6H_2_O → [UO_2_(H_2_O)_*y*_]_0.96_Sn_3.04_S_7.04_ + 1.92KNO_3_

For the Cs^+^ and Sr^2+^ exchanged samples the PXRD analysis showed a shift of the (020) and (040) basal Bragg peaks to lower 2*θ* values (higher *d*-spacing). The interlayer spacing of the material increases from 8.441 Å to 8.632 Å (Sr^2+^) and 8.813 Å (Cs^+^). The PXRD analysis of the UO_2_^2+^ exchanged sample shows the presence of a mixture of layered phases, which are mainly due to the different degrees of hydration of the UO_2_^2+^ ions. The interlayer spacing of the UO_2_^2+^ exchanged material increases from 8.441 Å to 9.966 Å and 10.250 Å. The change in the interlayer spacing follows the order UO_2_^2+^ > Cs^+^ > Sr^2+^ > K^+^, which is consistent with the ionic size of the ions. The TG analysis (Fig. S4†) showed that the degree of hydration for the exchanged materials follows the order Sr^2+^ > UO_2_^2+^ > Cs^+^ > K^+^.

**Fig. 5:**
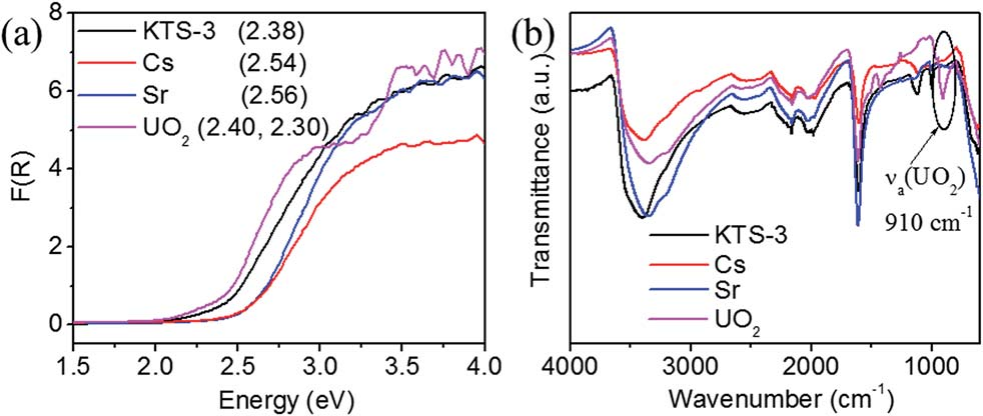
(a) Electronic absorption edges of KTS-3 (black), KTS-3 exchanged with Cs^+^ (red), Sr^2+^ (blue), and UO_2_^2+^ (magenta). The respective band gaps are 2.38, 2.54, 2.56, and 2.40, 2.30 eV, and (b) the IR spectra of KTS-3, Cs^+^, Sr^2+^ and UO_2_^2+^ exchanged material. The peak at ∼910 cm^–1^ corresponds to the antisymmetric vibration of the [O=U=O]^2+^ group.

The band gap of the pristine KTS-3 material is 2.38 eV and the yellow color of the material changes marginally upon exchange with Cs^+^ and Sr^2+^ ions. The exchanged materials show a small increase in absorption and the measured band gaps were 2.54 eV (Cs^+^) and 2.56 eV (Sr^2+^). With UO_2_^2+^ exchange, the yellow color slowly changes to a darker orange color and the band gaps red shifted to 2.30 eV and 2.40 eV (Fig. 5a). This can be attributed to partial dehydration of the UO_2_^2+^ ions and the presence of U···S interactions. The presence of two band gaps for the UO_2_^2+^ exchanged material was attributed to the differently hydrated UO_2_^2+^ ions.

The infra-red spectrum of the uranyl exchanged KTS-3 material shows a strong peak at ∼910 cm^–1^, which is not found in pristine KTS-3 (Fig. 5b). This peak at ∼910 cm^–1^ is assigned to the antisymmetric vibration of [O=U=O]^2+^ group and is significantly red shifted compared to the peaks found for aqueous [O=U=O]^2+^ ions (∼963 cm^–1^).^41^

**Fig. 6:**
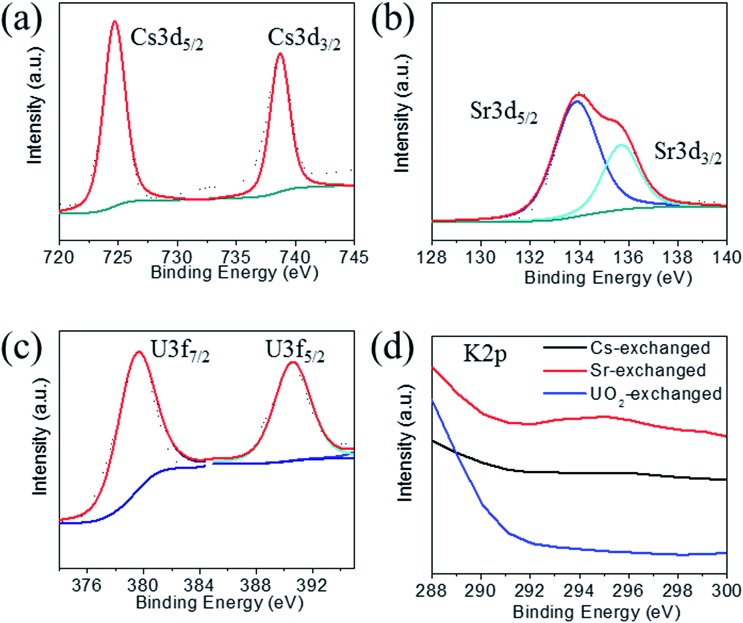
X-ray photoelectron spectra of (a) cesium, (b) strontium, (c) uranium and (d) potassium spectrum for the ion exchanged materials. Note that there was no peak observed for potassium for the exchanged materials, indication of complete exchange of potassium ions. Dotted and solid lines represent experimental and deconvoluted spectra, respectively.

The XPS spectra of the Cs^+^ exchanged samples show the characteristic 3d_5/2_ and 3d_3/2_ for Cs^+^ at 724.7 and 738.7 eV (Fig. 6a).^39^ The Sr^2+^ exchanged samples show peaks at 133.9 and 135.7 eV characteristic for 3d_5/2_ and 3d_3/2_ of Sr^2+^ cations (Fig. 6b).^39^ The UO_2_^2+^ exchanged samples show two peaks at 379.6 and 390.6 eV characteristic for 3f_7/2_ and 3f_5/2_ of U^6+^ centers (Fig. 6c).^31^,^39^ All exchanged samples showed the characteristic peaks for tin and sulfur ions as observed for the pristine compound. The peaks for the potassium 2p_3/2_ and 2p_1/2_ could not be found in the exchanged samples (Fig. 6d), which confirms their complete exchange from the KTS-3 compound.

### Ion exchange adsorption isotherm studies (Cs^+^, Sr^2+^ and UO_2_^2+^)

In order to understand the ion exchange capacity of KTS-3 a detailed adsorption study was carried out. The ion exchange equilibrium, kinetics, effect of salt concentration and pH on the Cs^+^, Sr^2+^ and UO_2_^2+^ ion exchange were studied. The equilibrium data for the ions were modeled using the Langmuir and Langmuir–Freundlich adsorption isotherms.^42^Table 2 shows the equilibrium constants and different parameters obtained by the modeling of the equilibrium data.

**Table 2:** The ion exchange sorption constants obtained by fitting the isotherm data with different models

	Cs^+^ ion exchange		Sr^2+^ ion exchange		UO_2_^2+^ ion exchange	
	Langmuir	Langmuir–Freundlich	Langmuir	Langmuir–Freundlich	Langmuir	Langmuir–Freundlich
*q*_e_ (mg g^–1^)	280(11)	304(22)	102 (5)	113(14)	287(15)	358(61)
*b* (L mg^–1^)	0.09(2)	0.07(2)	0.20(8)	0.19(15)	0.23(6)	0.09(6)
*n*	—	1.37(23)	—	1.81(54)	—	1.52(24)
*R* ^2^	0.965	0.972	0.923	0.928	0.952	0.960

Langmuir isotherm
4

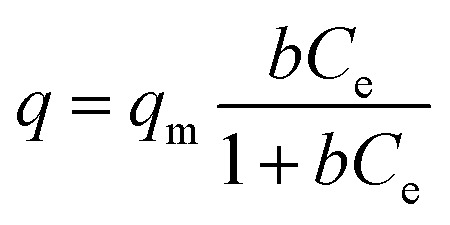




Langmuir–Freundlich isotherm
5

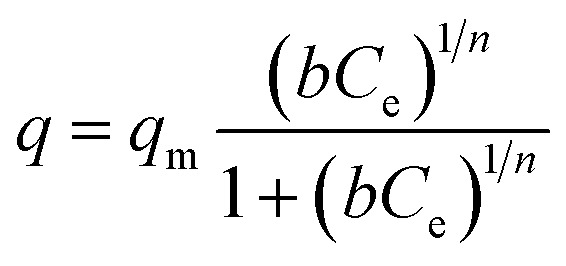

where *q* (mg g^–1^) is the amount of cation adsorbed at equilibrium concentration, *q*_m_ is the maximum cation adsorption capacity, *b* (L mg^–1^) is the Langmuir constant, *C*_e_ (ppm) is the equilibrium concentration and *n* is a constant.

The Langmuir isotherm describes adsorption on a homogenous surface and the maximum adsorption corresponds to a saturated monolayer. This model is based on the assumptions that (a) the adsorption sites are equivalent and each site can only accommodate one molecule, (b) the energy of adsorption is constant and independent of surface coverage, and (c) there is no transmigration of adsorbate from one site to another.^42^–^44^ The Langmuir–Freundlich isotherm is an extension of the Langmuir model, which reduces to Freundlich isotherms at low surface coverage and to Langmuir isotherms at high surface coverage.^42^

**Fig. 7:**
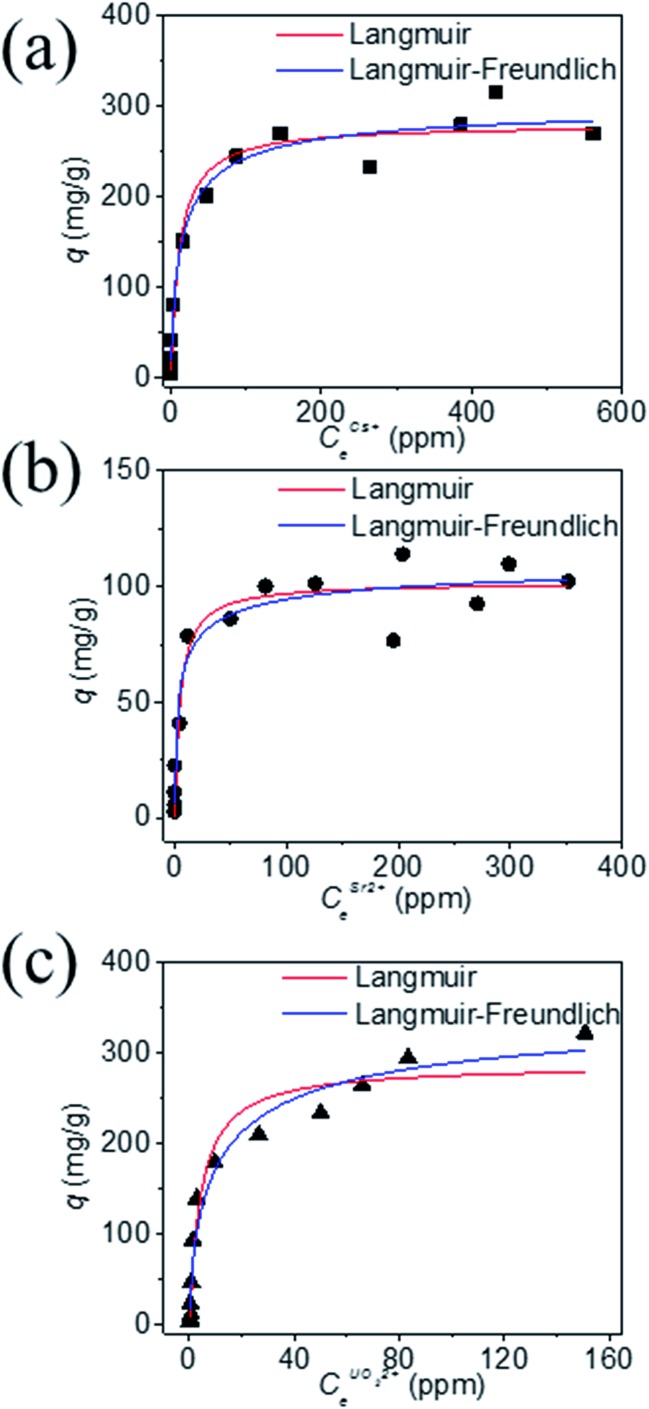
Equilibrium data for (a) Cs^+^, (b) Sr^2+^ and (c) UO_2_^2+^ ion exchange, the solid data represents the fitted lines by various isotherm models. The *V*/*m* ratio was 1000 mL g^–1^, pH ∼ 7 and the contact time was ∼15 h.

The equilibrium data for Cs^+^ ion exchange (Fig. 7a) could be fitted with both Langmuir, and Langmuir–Freundlich isotherm models with a good agreement (*R*^2^ ≥ 0.97). The value of the Langmuir–Freundlich constant *n* = 1.37(23) was found to be closer to 1 which suggests that the adsorption behavior of Cs^+^ ion exchange follows the Langmuir adsorption model. The agreement of the Langmuir adsorption isotherm with the Cs^+^ ion exchange can be rationalized by taking into consideration the structural features of KTS-3. The [Sn_3_S_7_]^2–^ layers of KTS-3 are separated by layers of disordered potassium ions, so the exchangeable Cs^+^ ions form a layer between the [Sn_3_S_7_]^2–^ layers that corresponds to the monolayer of Langmuir isotherms. The adsorption sites for the exchangeable ions are fixed (S^2–^ ions) and chemically equivalent. Moreover, once the ions are exchanged it is not possible to migrate to other sites. The equilibrium data for Sr^2+^ (Fig. 7b) and UO_2_^2+^ (Fig. 7c) were also fitted with Langmuir (*R*^2^ = 0.92 and 0.95, for Sr^2+^, UO_2_^2+^ respectively) and Langmuir–Freundlich adsorption (*R*^2^ = 0.92 and 0.96, for Sr^2+^, UO_2_^2+^ respectively) isotherms in good agreement. The value of Langmuir–Freundlich constant [*n* = 1.81(54) for Sr^2+^ and 1.52(24) for UO_2_^2+^] shows that it deviates from the Langmuir isotherm model (*n* = 1). The behavior of Cs^+^, Sr^2+^ and UO_2_^2+^ vis-a-vis their isotherms can be rationalized by the fact that the number of ions exchanged in the case of Cs^+^ is twice that of bivalent Sr^2+^ and UO_2_^2+^ and hence it has higher surface coverage and tends to follow better the Langmuir model. The ion exchange of bivalent metal ions often follow the Langmuir–Freundlich model rather than the Langmuir model.^45^

The maximum ion exchange capacities, *q*_m_ were found to be 280(11) mg g^–1^ (2.10 mmol g^–1^) for Cs^+^, 102(5) mg g^–1^ (1.16 mmol g^–1^) for Sr^2+^ and 287(15) mg g^–1^ (1.20 mmol g^–1^) for UO_2_^2+^ from the Langmuir isotherm model. The theoretical capacities for K_2*x*_Sn_4–*x*_S_8–*x*_ (*x* = 0.96) considering all the K^+^ ion are exchanged are 2.90 mmol g^–1^ (385 mg g^–1^) for Cs^+^ and 1.45 mmol g^–1^ for Sr^2+^ (127 mg g^–1^), UO_2_^2+^ (347 mg g^–1^). The observed Cs^+^ exchange is about 72%, Sr^2+^ exchange ∼ 80% and UO_2_^2+^ exchange ∼ 83% of the theoretical capacity. All K^+^ ions are exchanged after the reaction and the observed exchange capacity is due to the fact that the polycrystalline sample (K_2*x*_Sn_4–*x*_S_8–*x*_, KTS-3) has a range of *x* values from 0.65–1. The observed ion exchange capacity of KTS-3 compares well with well-known Cs^+^ and Sr^2+^ sorbents (*e.g.*, zeolites, sodium silicotitanates and zirconium titanium silicates; 1.86–4.1 mmol g^–1^ for Cs^+^ and 1.0–2.0 mmol g^–1^ of Sr^2+^).^46^–^49^

The Langmuir constants *b* (L mg^–1^) for the Cs^+^, Sr^2+^ and UO_2_^2+^ were found to be 0.09(2), 0.20(8) and 0.23(6) L mg^–1^, respectively. The value of *b* is an indicator for the affinity towards a particular ion. Higher *b* values for Sr^2+^ and UO_2_^2+^ ions indicate that KTS-3 has larger affinity towards them compared to Cs^+^. The affinity of a sorbent towards a particular ion can also be expressed in terms of distribution coefficient (*K*_d_),
6

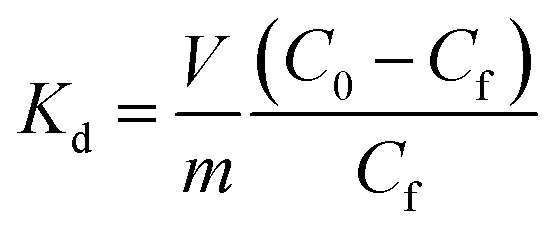

where, *V* is volume of testing solution (mL), *m* is the mass of the exchanger (g), *C*_0_ and *C*_f_ are the initial and final concentration of the ion.

The *K*_d_ values were found to be 5.5 × 10^4^ mL g^–1^ for Cs^+^, 3.9 × 10^5^ mL g^–1^ for Sr^2+^ and 2.7 × 10^4^ mL g^–1^ for UO_2_^2+^ (∼6–8 ppm, *V*/*m* = 1000 mL g^–1^ and pH = 7). *K*_d_ values in the 10^4^ or 10^5^ ranges are considered to be very good for ion exchange processes.^12^,^13^,^50^–^52^

### Ion exchange of Cs^+^ and Sr^2+^

Generally, nuclear waste contains a large amount of other non-radioactive ions (Na^+^, K^+^, Ca^2+^), also the waste solutions can be very corrosive depending on the pH.^53^ Ion exchange experiments with KTS-3 were performed over a range of pH and salt concentrations aimed to simulate the conditions likely to be found in nuclear waste treatment.

The stability of the KTS-3 phase over a range of pH values (2–12) was tested and was found to be impressive. The compound remains crystalline (3 ≤ pH ≤ 11) and retains the layered structure for days when suspended in solution. Even in highly acidic (pH ≤ 2) or basic conditions (pH ≥ 12) it remains stable for hours; a small decomposition of the compound can be seen if kept for more than 24 h (Fig. S5†).

**Fig. 8:**
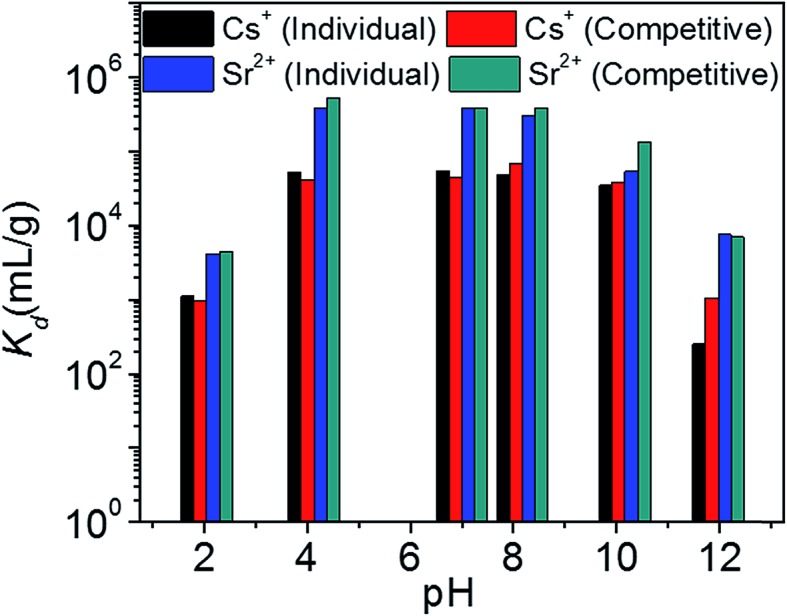
Variation of distribution coefficient *K*_d_ of individual and competitive Cs^+^ and Sr^2+^ ion exchange with increasing pH. The initial concentrations were 7.4 (Cs^+^) and 6.9 (Sr^2+^) ppm (both individual and competitive) and *V*/*m* ratio was 1000 mL g^–1^.

Fig. 8 represents the variation of *K*_d_ values for individual and competitive Cs^+^ and Sr^2+^ ion exchange with pH. KTS-3 shows excellent Cs^+^ ion exchange capacity over a pH range of 2–12. It absorbs over 97% of the ions from pH 4 to 10 and it absorbs around 53% of the ions even in a highly acidic environment (pH 2). The *K*Csd values were found to be ∼3.4 × 10^4^ to 5.5 × 10^4^ in the pH range of 4–10. However, there is slight decrease in the *K*Csd values at pH = 2 (1.1 × 10^3^) and it falls sharply at pH = 12 (253) (7.4 ppm, *V*/*m* = 1000). The decrease in *K*Csd values may be due to partial decomposition of KTS-3 in these regions of pH. The presence of Sr^2+^ in solution does not affect the ion exchange of Cs^+^, as the *K*Csd values found are comparable with those of the individual values (9.8 × 10^2^ to 6.7 × 10^4^). The small increase in *K*Csd in the presence of Sr^2+^ can be attributed to the overall increase in ionic charge of the solution.

KTS-3 exhibits remarkable Sr^2+^ capture capacity with more than 98% of the ions absorbed between pH 4 to 10. It decreases slightly at pH = 2 (81%) and 12 (88%) but is still much higher than Cs^+^. The *K*Srd values for the Sr^2+^ ion exchange over the pH range 2–12 were found to be 4.2 × 10^3^ to 3.9 × 10^5^ mL g^–1^ (6.9 ppm, *V*/*m* = 1000) (Fig. 8). The presence of Cs^+^ does not induce an appreciable change as the *K*Srd value remains almost same 4.5 × 10^3^ to 3.9 × 10^5^ mL g^–1^.

**Fig. 9:**
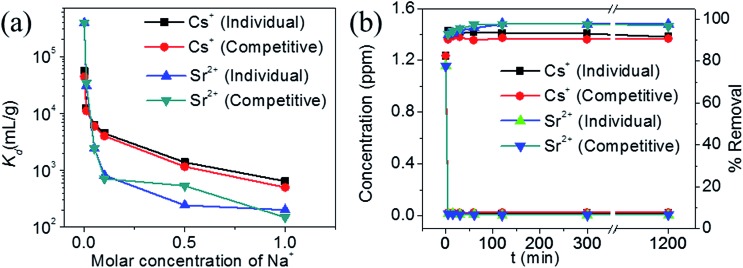
(a) Variation of distribution coefficient *K*_d_ of individual and competitive Cs^+^ and Sr^2+^ with increasing molar concentration of Na^+^ (initial concentrations of the ions were 7.4 (Cs^+^) and 6.9 (Sr^2+^) ppm, *V*/*m* ratio was 1000 mL g^–1^ and pH ∼ 7), and (b) kinetics of individual Cs^+^, Sr^2+^ and competitive ion-exchange *vs.* time *t* (min). The initial concentration was ∼1.2 ppm (for both Cs^+^ and Sr^2+^) and *V*/*m* ratio was 1000 mL g^–1^ and pH ∼ 7.

The *K*_d_ value for Cs^+^ in the presence of a huge excess of Na^+^ ions decreases slightly from 5.5 × 10^4^ mL g^–1^ at 0 M concentration to 4.4 × 10^3^ mL g^–1^ at 0.1 M concentration (Fig. 9a). Further increase in the Na^+^ concentration reduced the *K*_d_ values and even at a Na^+^ concentration of 1 M, KTS-3 exhibited a reasonable *K*_d_ value of 644 mL g^–1^. The *K*_d_ values in the presence of both Sr^2+^ and Na^+^ ions vary from 5.5 × 10^4^ mL g^–1^ at 0 M Na^+^ to 501 mL g^–1^ at 1 M Na^+^ concentration. The *K*_d_ value for Sr^2+^ in both individual and competitive (Cs^+^) ion-exchange reactions drops sharply in the presence of Na^+^. Namely, it decreases from 3.9 × 10^5^ mL g^–1^ at 0 M to 201 at 1 M Na^+^ concentration (Fig. 9a).

The kinetics of Cs^+^ adsorption for low concentration (∼1.2 ppm) solutions showed that 94% of the ions were absorbed within 5 min. The competitive Cs^+^ adsorption (in the presence of Sr^2+^) showed ∼90% adsorption within 5 min, which remains unchanged thereafter (Fig. 9b). The Sr^2+^ adsorption 92% (individual) and 92% (competitive in presence of Cs^+^) occurred within 5 min and with a longer time it increases to 97% (individual and competitive). The small decrease in ion exchange with time for Cs and increase for Sr^2+^ are due to the dynamic ion exchange process between K^+^ and Cs^+^/Sr^2+^ and the higher affinity of KTS-3 towards Sr^2+^. Upon contact with KTS-3, the Cs^+^ replaces the K^+^ ions immediately and only a small amount of K^+^ ions gets reabsorbed in the interlayer spaces to release some of initially absorbed Cs^+^ ions back to solution. However, in the case of Sr^2+^ the higher affinity of KTS-3 shuts down this dynamic ion exchange.

### Ion exchange of UO_2_^2+^

**Fig. 10:**
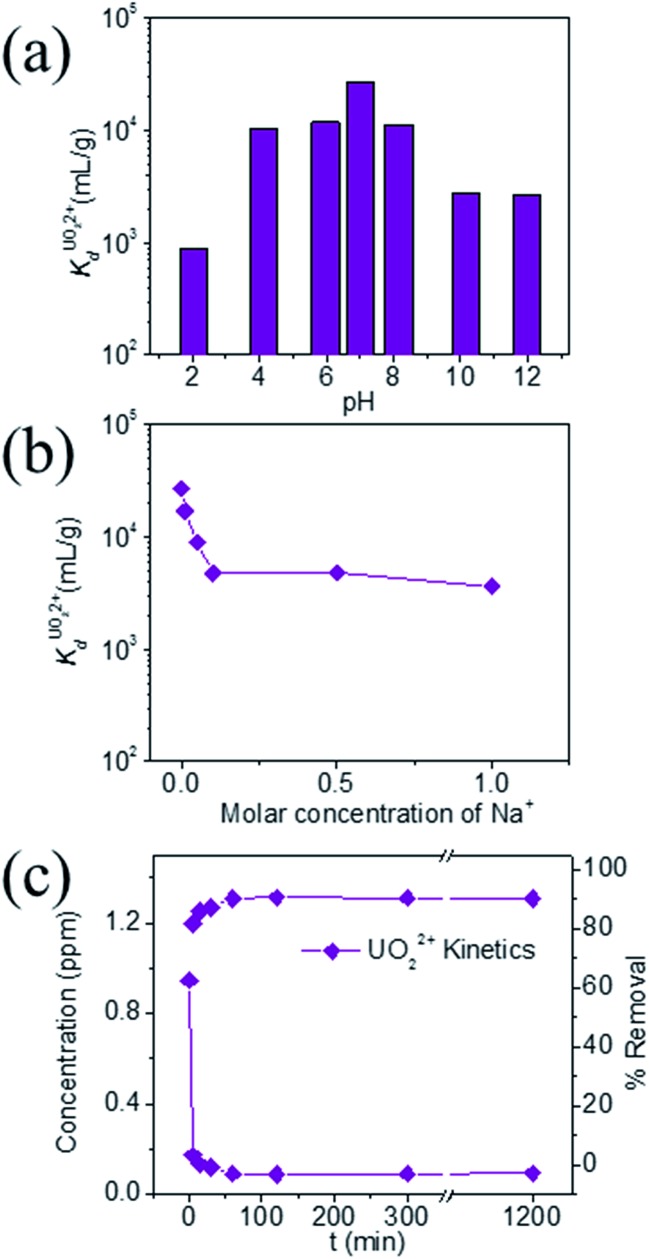
Variation of distribution coefficient 
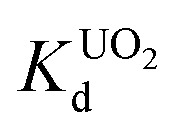
 (a) with pH, (b) increasing molar concentration of Na^+^. The initial concentration was 6 ppm, the *V*/*m* ratio was 1000 mL g^–1^ and (c) kinetics of individual UO_2_^2+^ ion-exchange *vs.* time (1 ppm, *V*/*m* ratio was 1000 mL g^–1^ and pH ∼ 7).

KTS-3 exhibits the best UO_2_^2+^ adsorption near neutral pH, the 
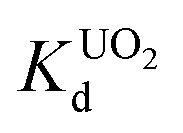
value at pH 7 is 2.7 × 10^4^ mL g^–1^. The UO_2_^2+^ adsorption capacity remains more or less the same between pH 4 to 8, however, it decreases at low (9.0 × 10^2^ mL g^–1^ at pH 2) and high (2.6 × 10^3^ mL g^–1^ at pH 12) pH values (Fig. 10a). The effect of Na^+^ on 
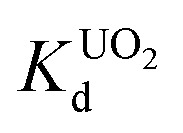
 is negligible; it decreases only slightly from 2.7 × 10^4^ mL g^–1^ at 0 M concentration to 4.8 × 10^3^ mL g^–1^ at 0.1 M concentration. Even at 1 M Na^+^ concentration the 
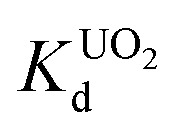
 is as high as 3.6 × 10^3^ mL g^–1^ (Fig. 10b). The kinetics of UO_2_^2+^ adsorption (∼0.95 ppm) solution showed that the ion exchange is rapid and 80% of the ions were adsorbed within 5 min, which increases to 90% with time (Fig. 10c). The UO_2_^2+^ ion exchange capacity of KTS-3 is good compared to other previously reported top UO_2_^2+^ sorbents.^31^,^33^,^54^–^56^

## Conclusions

The new compound K_2*x*_Sn_4–*x*_S_8–*x*_ (*x* = 0.65–1, KTS-3) has a unique anionic layer structure consisting of [SnS_6_] octahedra, [SnS_4_] tetrahedra, and long range ordered vacancies in the [SnS_4_]. The anionic layers are charge balanced by the interlayer potassium ions, which can be rapidly exchanged for Cs^+^, Sr^2+^ and UO_2_^2+^. KTS-3 exhibits high *K*_d_ values for Cs^+^ (5.5 × 10^4^), Sr^2+^ (3.9 × 10^5^) and UO_2_^2+^ (2.7 × 10^4^) ion exchange (7.4 ppm, 6.9, 5.7 ppm Cs^+^, Sr^2+^ and UO_2_^2+^, respectively; *V*/*m* ∼ 1000 mL g^–1^). The ion exchange capacity of the material remains mostly unaffected between pH 4–10 and decreases slightly in higher acidic or basic environment. The kinetics of the ion exchange showed that the process is very facile and it absorbs most of the ions within minutes.

The ion exchange capacity of K_2*x*_Sn_4–*x*_S_8–*x*_ (*x* = 0.65–1, KTS-3) is excellent and compares well with K_2*x*_M_*x*_Sn_3–*x*_S_6_ (M = Mn, KMS-1; M = Mg, KMS-2). The Cs and UO_2_^2+^ ion exchange capacity of KTS-3 (*q*_m_ = 226 mg g^–1^ for Cs^+^ and 382 mg g^–1^ for UO_2_^2+^) is comparable with KMS-1 (*q*_m_ = 280 mg g^–1^ for Cs^+^ and 287 mg g^–1^ for UO_2_^2+^) and the Cs^+^ ion exchange capacity is much lower than KMS-2 (*q*_m_ = 532 mg g^–1^ for Cs^+^). However, KTS-3 (*q*_m_ = 102 mg g^–1^ for Sr^2+^) outperforms both KMS-1 (*q*_m_ = 77 mg g^–1^ for Sr^2+^) and KMS-2 (*q*_m_ = 87 mg g^–1^ for Sr^2+^) in terms of Sr^2+^ ion exchange capacity. Moreover, the relative ease and inexpensive synthesis of K_2*x*_Sn_4–*x*_S_8–*x*_ make it a promising material for future studies.

Our work shows that the metal chalcogenide family can provide promising ion exchange materials for the selective removal of radionuclide from nuclear waste. Further work is to assess the utility of KTS-3 in remediation applications of nuclear wastes is justified.

## Author contributions

DS and MGK designed and conducted the research. The structure was solved by CDM. KSS and MSI helped characterizing the exchanged compounds by TGA, IR, Raman, UV-Vis and XPS. The manuscript was written by DS, CDM and MGK. All authors have approved the final version of the manuscript.

## Conflict of interest

The authors declare no competing financial interest.

## Supplementary Material

Supplementary information

Crystal structure data
