# Supplementary material for: K2xSn4–xS8–x (x = 0.65–1): a new metal sulfide for rapid and selective removal of Cs+, Sr2+ and UO22+ ions
Source: Chem Sci. 2015 Oct 27;7(2):1121–32. doi: 10.1039/c5sc03040d (PMC5975790; doi:10.1039/c5sc03040d)
Supplement: Supplementary file 1 [file SC-007-C5SC03040D-s001.pdf]

Electronic Supplementary Material (ESI) for Chemical Science.
This journal is © The Royal Society of Chemistry 2015

SUPPORTING INFORMATION

$K_{2x}Sn_{4-x}S_{8-x}$ (x=0.65-1): A New Metal Sulfide for Rapid and Selective Removal of Cs^+, Sr^{2+} and UO_2^{2+} ions

Debajit Sarma[†], *Christos D. Malliakas*[†], *K. S. Subrahmanyam*[†], *Saiful M. Islam*[†], *and Mercouri G. Kanatzidis*[*,†,‡]

[†]Department of Chemistry, Northwestern University, 2145 Sheridan Road, Evanston, IL 60208, USA

[‡]Materials Science Division, Argonne National Laboratory, Argonne, IL 60439, USA

E-mail: m-kanatzidis@northwestern.edu

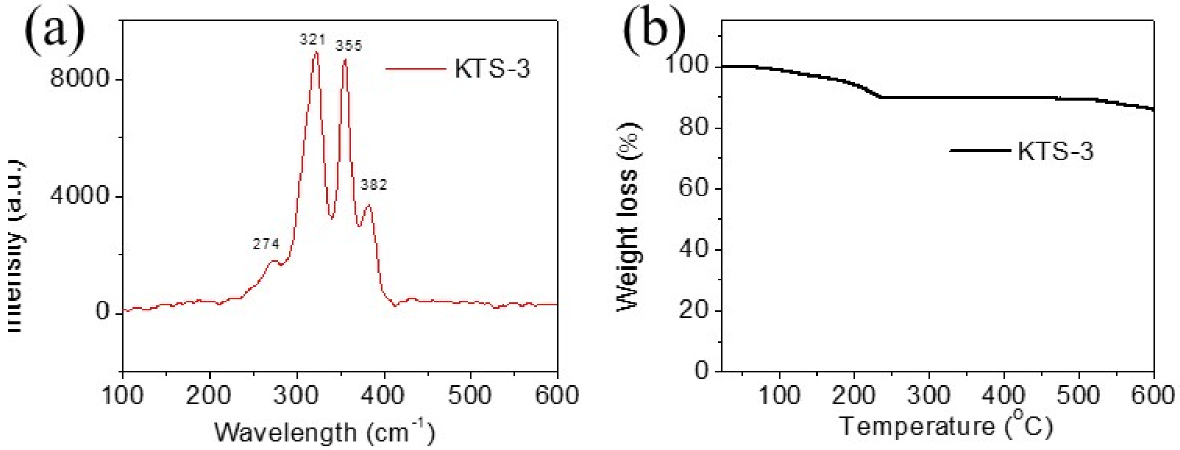

Figure S1. (a) Raman spectrum of KTS-3. The bands at 321, 355 are due to octahedral and the band at 382 cm^{-1} is due to tetrahedral Sn–S bond vibrations and (b) thermogravimetric analysis of KTS-3 compound. Initial weight loss of ~10% up to 235°C corresponds to the loss of adsorbed water molecules.

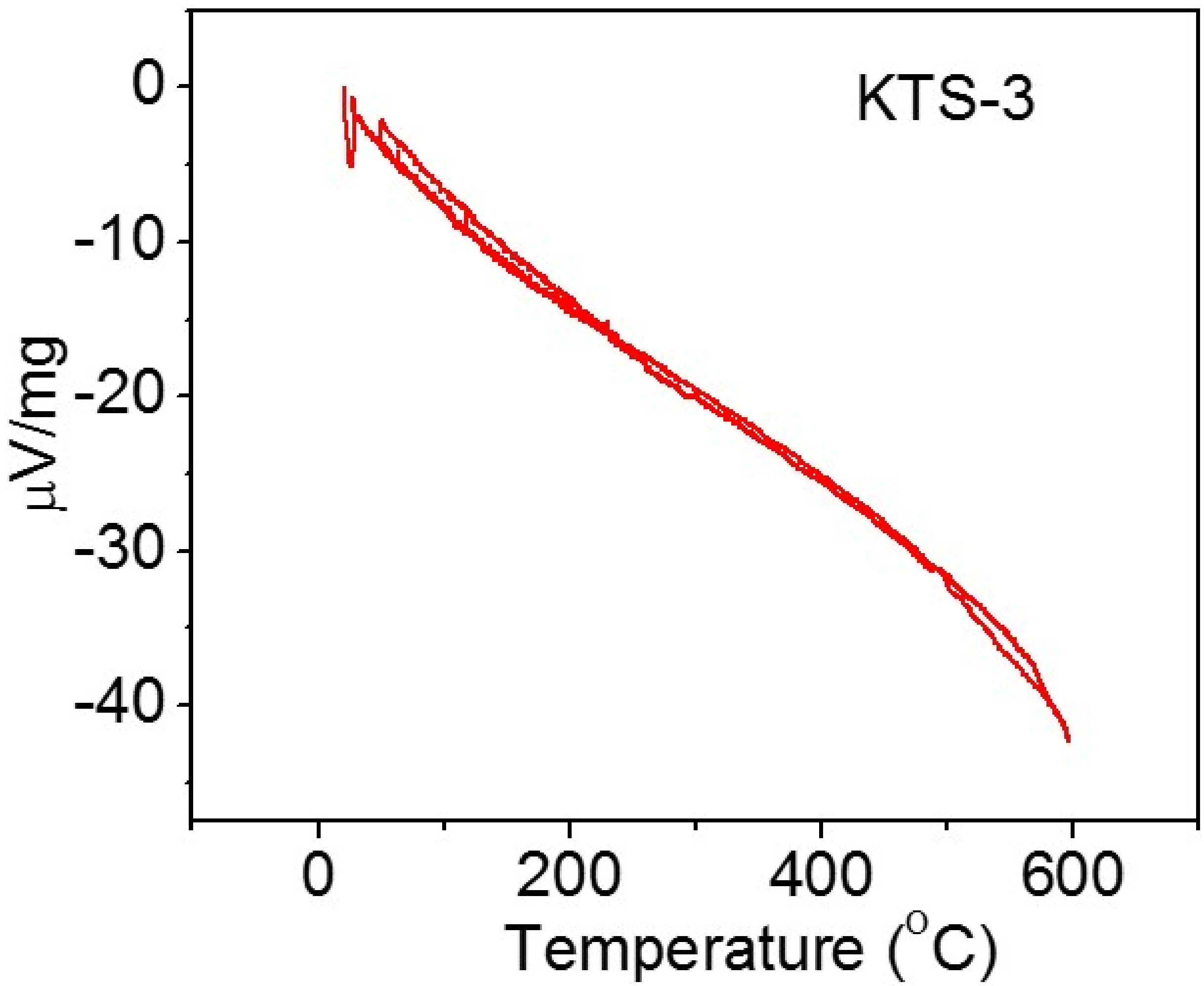

Figure S2. . Differential thermal analysis (DTA) of KTS-3 samples.

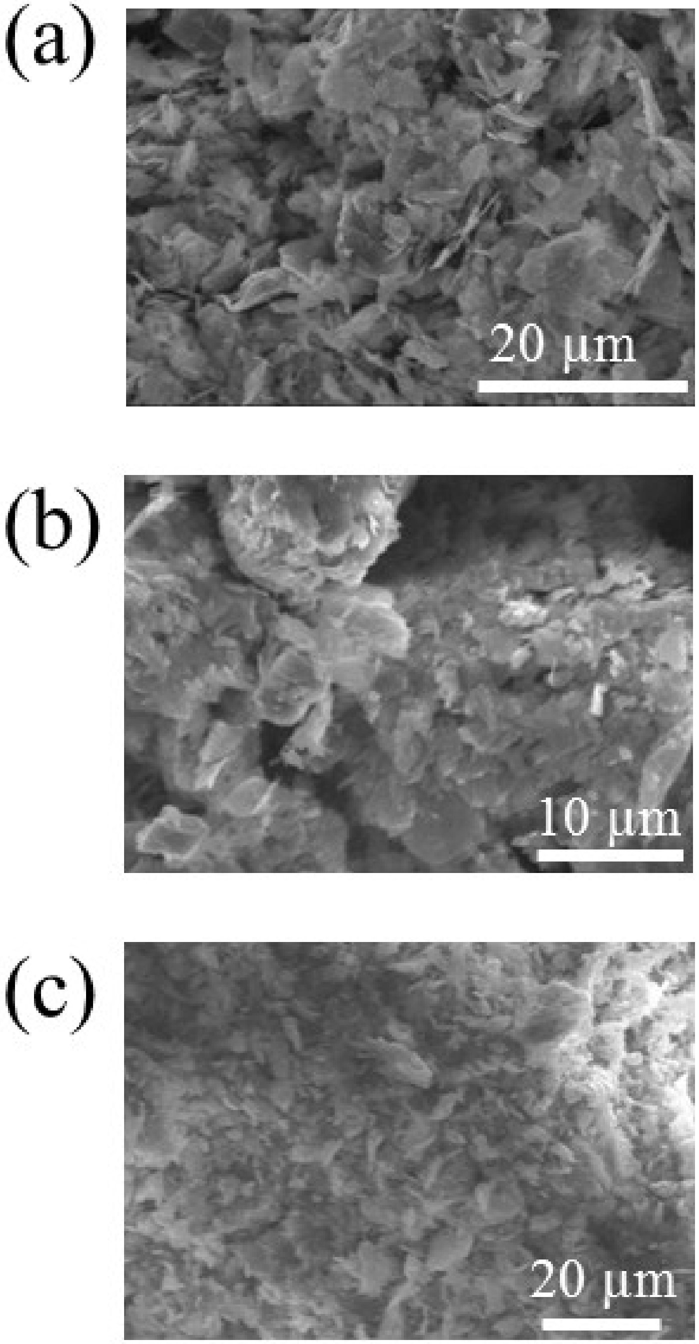

Figure S3. SEM image of the (a) Cs^{+}, (b) Sr^{2+}, and (c) UO_2^{2+} exchanged polycrystalline $K_{2x}Sn_{4-x}S_{8-x}$ (x=0.65-1, KTS-3) materials.

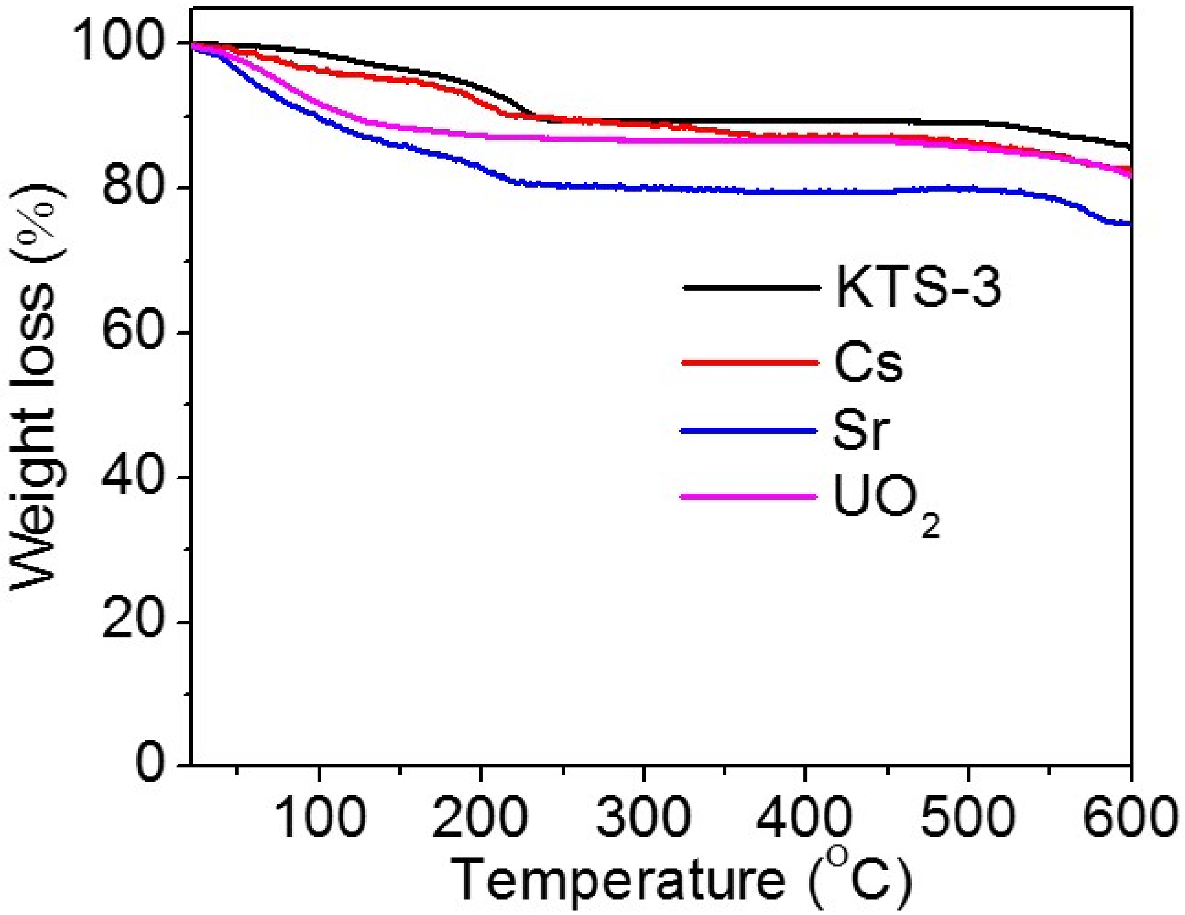

Figure S4. The TG analysis of the exchanged materials. Initial weight loss of ~10-20% up to 235°C corresponds to the loss of adsorbed water molecules. The degree of hydration for the exchanged materials follows the order Sr^{2+}> UO_2^{2+}> Cs^{+}>K^{+}.

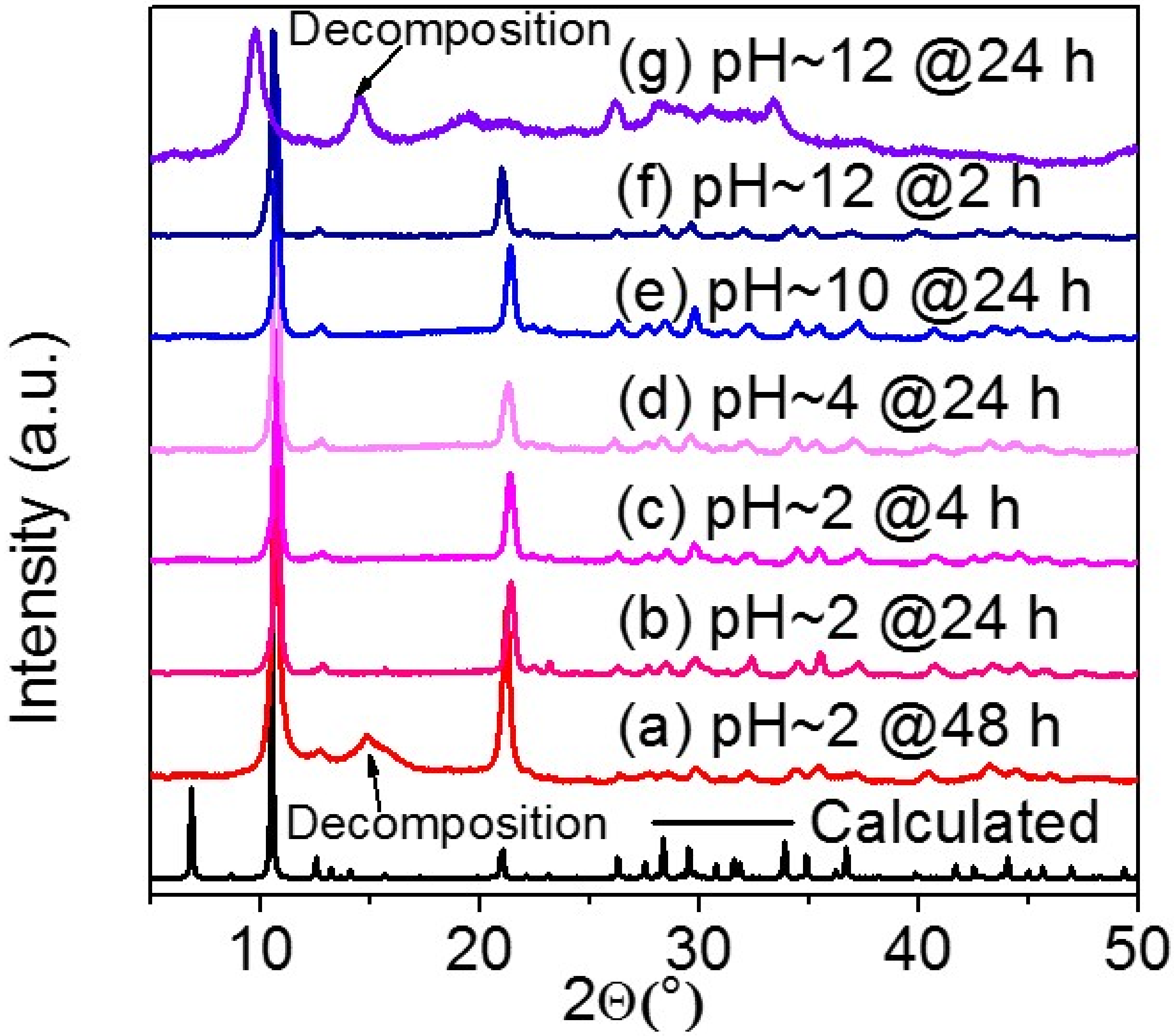

Figure S5. PXRD patterns of KTS-3 samples isolated at pH values of ~2, 4, 10, and 12.

Table 1: Fractional atomic coordinates and isotropic or equivalent isotropic displacement parameters (Å^2) for KTS-3- Subcell.

Atom	x	y	z	U_{iso}*/U_{eq}	Occupancy
Sn1	0.0000	0.18670 (3)	0.48613 (7)	0.0257 (4)	1.0
Sn2	−0.5000	0.06612 (8)	0.51825 (15)	0.0346 (6)	0.5
S1	0.0000	0.27693 (12)	0.4206 (2)	0.0244 (7)	1.0
S2	−0.5000	0.15494 (15)	0.3979 (3)	0.0343 (9)	1.0
S3	0.0000	0.10403 (15)	0.5675 (3)	0.0401 (10)	1.0
S4	−0.5000	0.0163 (3)	0.4038 (8)	0.053 (3)	0.5
K1	−0.5000	0.2438 (7)	0.2500	0.213 (11)	1.0
K2	0.0000	0.1628 (14)	0.7500	0.44 (3)	1.0

Table 2: Atomic displacement parameters (Å^2) for KTS-3- Subcell.

Atom	U^{11}	U^{22}	U^{33}	U^{12}	U^{13}	U^{23}
Sn1	0.0167 (6)	0.0185 (5)	0.0420 (7)	0.000	0.000	−0.0010 (3)
Sn2	0.0199 (10)	0.0203 (9)	0.0636 (15)	0.000	0.000	−0.0082 (8)
S1	0.0166 (15)	0.0211 (14)	0.0355 (16)	0.000	0.000	0.0001 (13)
S2	0.0188 (17)	0.0375 (19)	0.047 (2)	0.000	0.000	−0.0129 (16)
S3	0.045 (2)	0.0240 (16)	0.051 (2)	0.000	0.000	0.0072 (15)
S4	0.036 (5)	0.027 (4)	0.095 (8)	0.000	0.000	0.024 (4)
K1	0.40 (3)	0.175 (15)	0.067 (7)	0.000	0.000	0.000
K2	0.83 (10)	0.38 (4)	0.104 (14)	0.000	0.000	0.000

Table 3: Selected bond lengths (Å) for KTS-3- Subcell.

Sn1—S2	2.504 (3)
Sn1—S2^{i}	2.504 (3)
Sn1—S3	2.540 (4)
Sn1—S1	2.583 (3)
Sn1—S1^{ii}	2.596 (3)
Sn1—S1^{iii}	2.596 (3)
Sn2—S3^{iv}	2.245 (3)
Sn2—S3	2.245 (3)
Sn2—S4	2.316 (13)
Sn2—S4^{v}	2.504 (9)

Table 4: Selected bond angles (°) for KTS-3- Subcell.

S2—Sn1—S2^{i}	94.69 (14)
S2—Sn1—S3	92.44 (12)
S2^{i}—Sn1—S3	92.44 (12)
S2—Sn1—S1	92.52 (11)
S2^{i}—Sn1—S1	92.52 (11)
S3—Sn1—S1	172.67 (14)
S2—Sn1—S1^{ii}	177.44 (9)
S2^{i}—Sn1—S1^{ii}	87.45 (9)
S3—Sn1—S1^{ii}	88.87 (10)
S1—Sn1—S1^{ii}	85.97 (10)
S2—Sn1—S1^{iii}	87.45 (9)
S2^{i}—Sn1—S1^{iii}	177.44 (9)
S3—Sn1—S1^{iii}	88.87 (10)
S1—Sn1—S1^{iii}	85.97 (10)
S1^{ii}—Sn1—S1^{iii}	90.39 (11)
S3^{iv}—Sn2—S3	110.2 (2)
S3^{iv}—Sn2—S4	123.35 (12)
S3—Sn2—S4	123.35 (12)
S3^{iv}—Sn2—S4^{v}	100.3 (2)
S3—Sn2—S4^{v}	100.3 (2)
S4—Sn2—S4^{v}	87.7 (4)

Symmetry codes: (i) $x+1, y, z$; (ii) $-x+1/2, -y+1/2, -z+1$; (iii) $-x-1/2, -y+1/2, -z+1$; (iv) $x-1, y, z$; (v) $-x-1, -y, -z+1$.

Table 4: Fractional atomic coordinates and isotropic or equivalent isotropic displacement parameters (Å^2) for KTS-3 - Supercell.

Atom	*x*	*y*	*z*	U_{iso}*/U_{eq}	Occupancy
Sn1	0.12644 (7)	0.51425 (8)	0.1577 (2)	0.0310 (3)	1.0
Sn2	0.12670 (7)	0.51351 (8)	0.65342 (19)	0.0300 (3)	1.0
Sn3	0.36902 (10)	0.48174 (9)	−0.0319 (4)	0.0340 (4)	0.730 (4)
Sn4	0.3634 (3)	0.4812 (4)	0.4634 (13)	0.0734 (19)	0.311 (5)
S1	−0.0543 (3)	0.5785 (2)	0.1033 (6)	0.0291 (7)	1.0
S2	0.2926 (4)	0.4315 (3)	0.2125 (8)	0.0382 (11)	1.0
S3	0.2930 (4)	0.4338 (4)	0.6886 (10)	0.0478 (14)	1.0
S4	0.1865 (3)	0.6046 (2)	0.4258 (8)	0.0338 (7)	1.0
S5	0.1950 (3)	0.5996 (2)	−0.0756 (8)	0.0412 (9)	1.0
S6	0.0533 (2)	0.4205 (2)	0.3936 (6)	0.0278 (7)	1.0
S7	0.4706 (4)	0.5986 (4)	−0.0193 (13)	0.0473 (14)	0.730 (4)
S8	0.4534 (9)	0.5683 (9)	0.498 (3)	0.0473 (14)	0.311 (5)
K1A	0.0133 (9)	0.7525 (7)	0.412 (2)	0.101 (4)*	0.673 (17)
K1B	0.1665 (17)	0.2491 (13)	0.624 (3)	0.101 (4)*	0.419 (16)
K3	0.331 (4)	0.747 (4)	0.662 (11)	0.45 (3)*	0.82 (2)

Table 5: Atomic displacement parameters (Å^2) for KTS-3- Supercell.

Atom	U^{11}	U^{22}	U^{33}	U^{12}	U^{13}	U^{23}
Sn1	0.0249 (5)	0.0472 (7)	0.0218 (6)	−0.0019 (4)	0.0065 (9)	0.0000 (5)
Sn2	0.0235 (5)	0.0449 (6)	0.0214 (5)	0.0002 (4)	0.0020 (9)	0.0026 (5)
Sn3	0.0262 (5)	0.0532 (8)	0.0228 (6)	−0.0094 (5)	0.0045 (9)	0.0125 (8)
Sn4	0.031 (2)	0.133 (5)	0.062 (3)	−0.003 (2)	0.028 (3)	0.002 (5)
S1	0.0289 (15)	0.0401 (18)	0.0166 (14)	0.0029 (12)	−0.0031 (17)	0.0123 (17)
S2	0.031 (2)	0.051 (3)	0.035 (2)	0.005 (2)	0.016 (2)	0.015 (2)
S3	0.033 (2)	0.060 (4)	0.051 (3)	0.011 (2)	0.005 (3)	0.004 (3)
S4	0.0328 (15)	0.0453 (18)	0.0218 (15)	−0.0070 (13)	−0.001 (2)	0.002 (2)
S5	0.050 (2)	0.050 (2)	0.0230 (16)	−0.0175 (17)	0.004 (3)	0.009 (2)
S6	0.0215 (13)	0.0415 (18)	0.0198 (14)	−0.0032 (12)	0.0004 (18)	0.0087 (18)
S7	0.034 (2)	0.065 (3)	0.040 (3)	0.009 (2)	−0.007 (3)	0.015 (4)
S8	0.034 (2)	0.065 (3)	0.040 (3)	0.009 (2)	−0.007 (3)	0.015 (4)

Table 6: Selected bond lengths (Å) for KTS-3- Supercell.

Sn1—S5	2.505 (5)	Sn2—S4	2.483 (5)
Sn1—S4	2.533 (5)	Sn2—S5^{ii}	2.531 (6)
Sn1—S1^{i}	2.556 (4)	Sn2—S3	2.542 (5)
Sn1—S2	2.570 (5)	Sn2—S6	2.558 (4)
Sn1—S1	2.583 (4)	Sn2—S6^{iii}	2.586 (3)
Sn1—S6	2.630 (4)	Sn2—S1^{iii}	2.647 (4)
Sn3—S3^{iv}	2.305 (8)	Sn4—S8	1.879 (16)
Sn3—S2	2.341 (5)	Sn4—S2	2.122 (11)
Sn3—S7	2.374 (7)	Sn4—S3	2.163 (10)
Sn3—S7^{v}	2.483 (6)	Sn4—S8^{vi}	2.518 (13)

Table 7: Selected bond angles (°) for KTS-3- Supercell.

S5—Sn1—S4	95.04 (14)	S4—Sn2—S5^{ii}	93.92 (13)
S5—Sn1—S1^{i}	88.02 (15)	S4—Sn2—S3	92.84 (18)
S4—Sn1—S1^{i}	176.02 (13)	S5^{ii}—Sn2—S3	91.2 (2)
S5—Sn1—S2	92.51 (15)	S4—Sn2—S6	89.67 (14)
S4—Sn1—S2	92.40 (17)	S5^{ii}—Sn2—S6	176.41 (14)
S1^{i}—Sn1—S2	90.00 (16)	S3—Sn2—S6	88.72 (18)
S5—Sn1—S1	93.49 (14)	S4—Sn2—S6^{iii}	90.62 (13)
S4—Sn1—S1	92.59 (14)	S5^{ii}—Sn2—S6^{iii}	94.14 (15)
S1^{i}—Sn1—S1	84.68 (14)	S3—Sn2—S6^{iii}	173.42 (18)
S2—Sn1—S1	171.83 (15)	S6—Sn2—S6^{iii}	85.70 (14)
S5—Sn1—S6	177.93 (16)	S4—Sn2—S1^{iii}	176.91 (13)
S4—Sn1—S6	87.00 (14)	S5^{ii}—Sn2—S1^{iii}	85.54 (14)
S1^{i}—Sn1—S6	89.96 (12)	S3—Sn2—S1^{iii}	90.21 (18)
S2—Sn1—S6	87.00 (14)	S6—Sn2—S1^{iii}	90.87 (12)
S1—Sn1—S6	86.81 (12)	S6^{iii}—Sn2—S1^{iii}	86.39 (12)
S3^{iv}—Sn3—S2	112.58 (16)	S8—Sn4—S2	128.1 (8)
S3^{iv}—Sn3—S7	119.7 (3)	S8—Sn4—S3	120.7 (8)
S2—Sn3—S7	124.7 (3)	S2—Sn4—S3	110.0 (3)
S3^{iv}—Sn3—S7^{v}	101.2 (3)	S8—Sn4—S8^{vi}	71.0 (7)
S2—Sn3—S7^{v}	97.4 (2)	S2—Sn4—S8^{vi}	104.9 (6)
S7—Sn3—S7^{v}	89.5 (2)	S3—Sn4—S8^{vi}	107.0 (6)

Symmetry codes: (i) –x, -y+1, -z; (ii) x, y, z+1; (iii) –x, -y+1, -z+1; (iv) x, y, z-1; (v) –x+1, -y+1, -z; (vi) –x+1, -y+1, -z+1.
